# Accelerating Nature: Induced Atomic Order in Equiatomic FeNi

**DOI:** 10.1002/advs.202302696

**Published:** 2023-12-10

**Authors:** Laura H. Lewis, Plamen S. Stamenov

**Affiliations:** ^1^ Department of Chemical Engineering and Department of Mechanical and Industrial Engineering Northeastern University Boston MA 02115 USA; ^2^ School of Physics & CRANN Trinity College Dublin 2 Ireland

**Keywords:** chemical order, meteorites, Mössbauer spectroscopy, permanent magnets, phase transitions

## Abstract

The production of locally atomically ordered FeNi (known by its meteoric mineral name, tetrataenite) is confirmed in bulk samples by simultaneous conversion X‐ray and backscattered γ‐ray ^57^Fe Mössbauer spectroscopy. Up to 22 volume percent of the tetragonal tetrataenite phase is quantified in samples thermally treated under simultaneous magnetic‐ and stress‐field conditions for a period of 6 weeks, with the remainder identified as the cubic FeNi alloy. In contrast, all precursor samples consist only of the cubic FeNi alloy. Data from the processed alloys are validated using Mössbauer parameters derived from natural meteoritic tetrataenite. The meteoritic tetrataenite exhibits a substantially higher degree of atomic order than do the processed samples, consistent with their low uniaxial magnetocrystalline anisotropy energy of ≈1 kJ·m^−3^. These results suggest that targeted refinements to the processing conditions of FeNi will foster greater atomic order and increased magnetocrystalline anisotropy, leading to an enhanced magnetic energy product. These outcomes also suggest that deductions concerning paleomagnetic conditions of the solar system, as derived from meteoritic data, may warrant re‐examination and re‐evaluation. Additionally, this work strengthens the argument that tetrataenite may indeed become a member of the advanced permanent magnet portfolio, helping to meet rapidly escalating green energy imperatives.

## Graphical abstract

Processing‐induced atomic order is confirmed in FeNi alloys and compared to that of its meteoritic analog, tetrataenite, using simultaneous conversion X‐ray and backscattered γ‐ray ^57^Fe Mössbauer spectroscopy. The concurrent application of saturating magnetic field and strain applied during 6 weeks of thermal treatment creates up to 22 vol.% of the ordered phase, of interest for future advanced permanent magnets.

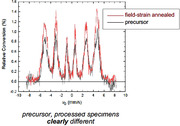

## 1 Introduction

Nature is the primary inspiration, as well as the ultimate engineer, of materials for technological application. However, some compounds and structures, while thermodynamically accessible, remain kinetically inaccessible and require natural formation timescales that are so lengthy that they are essentially unobtainable on Earth. An associated corollary is that such materials, if they do indeed exist on Earth, might be present in such minute quantities or arranged in hidden locations so as to escape detection unless highly specific probes and hypotheses regarding their environment are deployed. Both of these scenarios are relevant to investigations of the unique extraterrestrial mineral “tetrataenite”, which is under consideration as a next‐generation, sustainable permanent magnet, provided that it can be produced with sufficient quality in industrially relevant timescales. Addressing this possibility, this current work reports ^57^Fe Mössbauer spectroscopy data obtained from a selection of specially processed FeNi alloys confirming the terrestrial production of locally ordered tetrataenite, up to 22 vol%, in a time period of only 6 weeks. These results are validated by data collected in parallel from large natural tetrataenite regions within the extremely slowly cooled metallic Ni‐rich meteorite NWA 6259. The processed sample phases are found to possess a lower degree of atomic order than that present in the meteorite, a conclusion supported by larger Mössbauer absorption peak widths as well as by magnetic data that return a low uniaxial magnetocrystalline anisotropy value (≈1 kJ·m^−3^) for the processed samples. It is hypothesized that refinements to both the composition and to the processing conditions of FeNi alloys will foster increased induced atomic order, increased magnetocrystalline anisotropy and an enhanced magnetic energy product. Overall, these results strengthen the argument that tetrataenite may be considered as a member of the advanced permanent magnet portfolio, helping to meet rapidly escalating green energy imperatives. At the same time these results suggest an opportunity for a revisited perspective of celestial metallurgy and associated interpretations of origins of our solar system.

## 2 Background

### 2.1 Tetrataenite's Place in the Permanent Magnet Portfolio

Permanent magnets are essential to modern society, allowing interconversion and storage of mechanical and electrical energy to perform work and enable 21st‐century aspirations for e‐mobility, robots, and drones. At the same time, systems utilizing advanced magnetic materials permit energy harvesting from wind, water, and wave sources, mitigating dependence on carbon‐based fuels. While high‐performance permanent magnets comprised of platinum group/rare‐earth element compounds including FePt, Nd_2_Fe_14_B, and SmCo_5_ are excellent for such applications, these materials are either entirely too expensive, too rare and/or are geopolitically constrained for continued widespread deployment, with demand predicted to outstrip supply within a decade.^[^ ^1^ ^]^ This scenario provides strong motivation to develop new types of magnetic materials that are free of constrained “critical” elements^[^ ^2^, ^3^ ^]^ and sets the stage for re‐examining tetrataenite, the mineral name for atomically ordered (L1_0_) FeNi, as an advanced permanent magnet.

**Figure 1:**
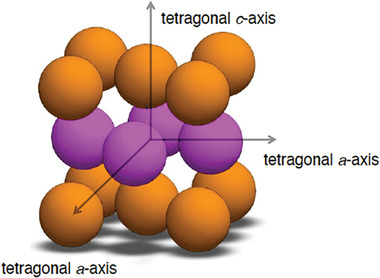
The L1_0_ atomically ordered crystal structure of tetrataenite.

Tetrataenite possesses a nominally equiatomic FeNi composition and is reported to exist naturally only in nickel‐rich, stony, stony‐iron, and iron meteorites that have been subjected to extremely slow cooling rates, in the range 0.2–10 000 kelvins per 10^6^ years.^[^ ^4^, ^5^ ^]^ This mineral, which has escaped documentation on standard engineering‐based binary Fe–Ni phase diagrams,^[^ ^6^, ^7^ ^]^ is distinguished from earthly FeNi alloys in that each of its two elemental constituents occupies specific atomic lattice sites in a tetragonal crystal lattice. In other words, tetrataenite is not an alloy featuring random lattice site occupancies but is rather an atomically ordered intermetallic compound with the constituent Fe and Ni atoms forming a superlattice of atomic layers. These layers are oriented along the tetragonal *c*‐axis, which is also the direction of spontaneous magnetization. Employing the Strukturbericht classification of crystal structure types, this lattice adopts the L1_0_‐type crystal structure (see **Figure** **1**). Perfectly atomically ordered structures may be assigned an order parameter ***S*** = 1 that can decrease monotonically to a value of ***S*** = 0 that describes fully randomized lattice site occupancy. Depending upon the degree of atomic order, these atomic layers confer L1_0_‐type structures with unique mechanical, electronic, and magnetic properties. Crucially, the very high magnetocrystalline anisotropy energy of well‐known L1_0_‐type ferromagnetic compounds such as CoPt and FePt is strongly dependent upon the degree of atomic order present in the lattice.^[^ ^8^, ^9^, ^10^ ^]^ The magnetocrystalline anisotropy, an intrinsic parameter that describes how strongly the magnetization vector is locked into certain crystallographic directions, limits the achievable coercivity of a magnet, which is the relevant engineering parameter that describes a magnet's resistance to demagnetization.

First observed in the mid‐1970′s in the Cape York, Toluca and Santa Catharina iron meteorites,^[^ ^11^, ^12^, ^13^ ^]^ tetrataenite is a component of the famous Widmanstätten pattern that is comprised of long interwoven nickel‐iron crystals in which the close‐packed planes of the nickel‐poor body‐centered cubic (bcc) form of iron (*aka* kamacite) form on the close‐packed planes of the nickel‐rich face‐centered‐cubic (fcc) form of iron (*aka* taenite).^[^ ^14^ ^]^ While tetrataenite was formally identified as a new meteoritic mineral in 1980,^[^ ^15^ ^]^ it attracted significant technological interest some thirty years later, during the so‐called “Rare Earth Crisis”^[^ ^16^ ^]^ that first escalated in 2012. During that time tetrataenite was contemplated as an advanced permanent magnetic material that was entirely free of constrained or critical elements, if synthesis and processing paths could be developed to realize it in industrially relevant timescales. Today, tetrataenite remains an active topic of discussion within the permanent magnet community. While development of a permanent magnet based on tetrataenite is widely considered to be technologically disruptive, to date no R&D effort has been successful in synthesizing this compound, in a form useful for permanent magnet applications. Summaries of efforts and reported outcomes to synthesize tetrataenite may be found in refs. [17, 18]

The potential of a given magnetic material to become a useful permanent magnet is dependent upon the energy density that the material can store as well as on its magnetic transition temperature. This energy density, which may be quantified as the maximum energy product (*BH*_max_) where *B* is the magnetic flux density and *H* is the magnetic field strength, is dependent in part upon the material's magnetocrystalline anisotropy energy, which must be sufficiently high to withstand self‐demagnetization.^[^ ^19^ ^]^ These engineering parameters may be critically examined in tetrataenite. While there is general agreement in the magnetism community that tetrataenite possesses an appropriately high magnetic flux density appropriate for high‐quality permanent magnets,^[^ ^17^ ^]^ less consensus exists regarding the magnitude of its magnetocrystalline anisotropy energy.^[^ ^19^ ^]^ The scientific record contains only a handful of historical^[^ ^20^ ^]^ or more recent reports of experimentally determined magnetocrystalline anisotropy energy values of ordered FeNi.^[^ ^21^, ^22^, ^23^ ^]^ In a few of these reports the degree of atomic order ***S*** was also disclosed: for example, an anisotropy energy value of 7 kJ·m^−3^ and atomic order parameter of ***S*** = 0.48 were reported for an 18‐nm‐thick FeNi film^[^ ^21^ ^]^ while an anisotropy energy value of 9 kJ·m^−3^ and an order parameter of ***S*** = 0.2 were measured from a layered film comprised of 50 monolayers each of Fe and Ni.^[^ ^24^ ^]^ The highest reported value of coercivity in natural (meteoritic) tetrataenite is 4000 Oe (318 kA m^−1^),^[^ ^25^ ^]^ obtained from an obviously unoptimized source. Overall, literature that reports both the anisotropy energy and the corresponding atomic order are very rare, likely due to the scarcity of natural tetrataenite as well as to its aforementioned synthesis challenges. Finally, it is known that the magnetization of tetrataenite may be kinetically stabilized to high temperatures, to at least 500 °C, with a measured temperature coefficient of coercivity of −0.005% K^−1^, a value over one hundred times smaller than that of Nd_2_Fe_14_B‐based magnets.^[^ ^26^ ^]^ As the relationships between magnetocrystalline anisotropy energy, atomic order, and magnetic disordering behavior of tetrataenite are not yet settled topics, this material may continue to be considered as a candidate hard magnetic material suited for so‐called “gap magnet” applications.^[^ ^27^ ^]^

### 2.2 Inducing and Detecting Atomic Order in FeNi

Developing atomic order in FeNi alloys to form tetrataenite requires review of its development in nature. Upon cooling from elevated temperatures, long‐range atomic ordering in FeNi is reported to take place ≈320 °C,^[^ ^28^, ^29^, ^30^ ^]^ transforming the high‐temperature disordered fcc FeNi alloy into the more thermodynamically stable tetragonal L1_0_ compound. Adapting this extraterrestrial process, Lewis and coworkers subjected highly strained FeNi‐based specimens to a 290 °C vacuum annealing process for 24–30 days with the intention to activate L1_0_ phase transformation.^[^ ^31^ ^]^ Results from these conventionally annealed specimens confirmed attainment of tetragonality but no L1_0_ superstructure was detected.^[^ ^31^ ^]^ Accordingly, these data allowed the hypothesis that the A1‐L1_0_ phase transformation in bulk FeNi may be promoted by two consecutive operations: i) applied stress to induce a martensitic (i.e., diffusionless) transformation from the cubic structure to an intermediate, atomically disordered tetragonal structure (A6 Strukturbericht designation) followed by ii) a nucleation/diffusional process to induce atomic order and thereby amplify the magnetocrystalline anisotropy. Connecting these processes to the Gibbs Energy formalism of phase transformations,^[^ ^32^, ^33^ ^]^ it was proposed that the introduction of mechanical stress and magnetic field during annealing will favorably tip thermodynamic equilibria as well as lower kinetic barriers, thus encouraging atomic ordering. The capability to include these additional energy contributions was incorporated into a custom‐built furnace that can deliver a passive, uniform saturating magnetic field along with tensile stress, appropriate for long‐time annealing protocols.^[^ ^34^, ^35^ ^]^ Although the proposed route to accelerate atomic ordering in equiatomic FeNi may be conceptually clear, confirmation and quantification of its effects is extraordinarily challenging. In general, X‐ray, neutron and/or electron diffraction probes can register the degree of atomic order in a crystal structure only if sufficient differences in atomic or nuclear scattering cross sections of the constituent atoms are present.^[^ ^36^, ^37^ ^]^ As iron and nickel share very similar scattering cross sections, originating from their similar electronic structures, only extremely weak L1_0_ superlattice signatures can be produced, even in the case of perfect atomic order. To wit, calculated diffraction patterns modeling a 100% volume fraction of perfectly ordered (***S*** = 1) L1_0_‐type FeNi return ratios of intensities of the strongest superstructure (001) diffraction peak to that of the main fundamental (111) diffraction peak of only 0.0030:1 for X‐rays and an even smaller ratio 0.0028:1 for neutrons.^[^ ^38^ ^]^ In other words, the maximum superlattice peak intensity is more than 3000 times smaller than the fundamental peak intensity. This issue is further exacerbated in specimens containing small and/or poorly ordered L1_0_ phase fractions, as well as in those that might contain easily produced iron‐based oxides that contribute strong diffraction peaks overlying the L1_0_ FeNi superlattice peaks.^[^ ^39^, ^40^ ^]^ Moreover, sample surface preparation of FeNi specimens is of utmost importance for accurate characterization: particular attention must be paid to the polishing procedure to avoid creation of a strained surface layer that damages and distorts the surface magnetic domain configuration.^[^ ^34^, ^41^, ^42^ ^]^ Earlier (unpublished) results from the current authors confirm that FeNi‐based samples can exhibit highly complex magnetic domain patterns of uniaxial, out‐of‐plane magnetic polarization that are actually artifacts of mechanical polishing.

In contrast to diffraction approaches, Mössbauer spectroscopy is one of the very few techniques that can directly examine the local atomic order in FeNi. Mössbauer spectroscopy is a well‐established probe for the study of many types solid materials (especially those that contain iron) and has in particular been widely applied to the characterization of iron meteorites.^[^ ^43^ ^]^ In this current work, local atomic order in FeNi alloys that was induced by thermal processing under simultaneous stress and magnetic field conditions was probed and confirmed using the unique technique of simultaneous conversion X‐ray and backscattered γ‐ray ^57^Fe Mössbauer spectroscopy (CXMS). The CXMS approach, which is far less prolific than transmission mode Mössbauer spectroscopy, utilizes recoilless nuclear resonant absorption of gamma rays to allow good resolution of hyperfine interactions that document the local magnetic and atomic environments of iron nuclei. In contrast to conventional Mössbauer spectroscopy, CXMS can often be performed with little or no sample surface preparation and allows for selective depth profiling on the scale of 1–30 µm, courtesy of the different absorption lengths of the characteristic 6.4 keV X‐ray and 14.4 keV γ‐ray photons. In this manner small changes in the resonant absorption energies allow quantification of electron densities (isomer shift), distortions in the local crystal environment (quadrupole splitting) and magnetic order arising from both nuclear and non‐nuclear contact interaction magnetic fields (Zeeman splitting), as measured by the non‐zero electron density (of typically s‐state electrons) at the probe nuclei.^[^ ^44^ ^]^ While Mössbauer spectroscopy fosters phase identification through determination of bond character, spin state, oxidation state and magnetic interactions, depending on the specific parameters that are extracted, it provides no high‐resolution information on the spatial extent or distribution of the detected phases. However, refined Mössbauer parameters obtained by fitting the experimental data can provide information concerning the character of the very local iron nuclear environment. In particular, the energy levels of the nuclei of an iron atom in a magnetically ordered state split to allow six possible nuclear transitions, resulting in a sextet, or non‐zero, hyperfine field (*B*_hf_) absorption spectrum. The widths of the hyperfine absorption peaks are an indication of the distribution of nearest neighbors or site occupancy of iron in a given site and thus provide information on the degree of local atomic order and/or crystallinity. In this manner local atomic environments of greater disorder correspond to wider outer peaks of the obtained sextet pattern.

In this work CXMS was performed in backscattering mode to yield two simultaneously acquired data products: an X‐ray (6.4 keV) Mössbauer spectrum and a backscattered γ‐ray spectrum (14.4 keV). As these photons have absorption lengths in the range 5–50 µm, depending on angle of incidence and elemental (isotopic) composition of the sample, they may be used for the characterization of the surface regions of larger bulk samples, while not suffering from the excessive surface sensitivity, associated with conversion electron Mössbauer spectroscopy (CEMS). These attributes allow CXMS to bypass deleterious artifacts such as surface irregularities and induced strain resulting from sample preparation that can impact the quality of collected data. Thus, CXMS can be more useful than other types of Mössbauer spectroscopies, such as conventional (CEMS) with a surface sensitivity in the range 5–500 nm or conventional transmission‐mode Mössbauer spectroscopy that is limited to the examination of thin (<20–100 µm) or samples that are dilute in iron.

## 3 Results

### 3.1 Synopsis

The natural tetrataenite sample from the NWA 6259 meteorite is confirmed to contain both the atomically ordered tetragonal L1_0_ phase as well as the disordered cubic Al phase, along with a significant fraction of a third unidentified phase. No other information concerning the nature of this third unidentified phase is available at this time. Mössbauer parameters were obtained from the meteorite spectra and applied to spectra measured from the FeNi alloy strips. Analysis of these spectra allowed the determination that all magnetic‐field‐/stress‐field‐processed (FSA) samples consist of ≈20 vol.% of the atomically ordered L1_0_ phase along with ≈80 vol.% of the disordered A1 phase, while all precursor samples are comprised of 100 vol.% A1 (fcc) atomically disordered phase. No other phases are found in the FeNi strips. These results are summarized in **Table** **1**.

**Table 1:** Summary of sample identity, processing conditions and phase constitutions. “FSA” identifies the processing condition of annealing under simultaneous applied magnetic field and stress conditions.

NWA 6259 meteorite	Phase fractions [vol.%]: L1_0_–65 – 72%; fcc (A1)–15–16 vol. %; unidentified paramagnetic phase–13–19 vol.%, depending on probing depth in the specimen.		
Synthesized sample composition (atomic basis)	Annealing condition	Phase fractions [vol.%]	
		fcc phase (A1 Strukturbericht designation)	Atomically ordered phase (L1_0_ Strukturbericht designation)
Fe^62^Ni	FSA	78% [+/− 17%]	22% [+/−10]
(Fe^62^Ni)_98_Ti_2_	FSA	83% [+/− 17%]	17% [+/− 10]
(FeNi)_98_Nb_2_	FSA	79% [+/− 17%]	21% [+/− 10]
(FeNi)_98_Mo_2_	FSA	81% [+/− 17%]	19% [+/− 10]
(FeNi)_98_Mo_2_	Cold‐rolled precursor	100%	—
(FeNi)_98_Ti_2_	Cold‐rolled precursor	100%	—

Magnetic characterization of the processed samples did not reveal any magnetic in‐plane anisotropy above the threshold of detection, equivalent to ≈1 kJ·m^−3^ of equivalent uniaxial anisotropy energy. A small piece of NWA 6259 was determined to possess a large moment of ≈5.3 kA·m^2^ at 300 K. This piece was measured after zero‐field cooling as well as after field‐cooling in a field of *µ*_0_ *H* = 5 T to yield a coercivity of *µ*_0_ *H*_C_ ≈0.1 T at room temperature. No exchange bias was observed in the meteoritic specimen at low temperature, and a moment freezing of less than 10% of the remanence value was obtained.

### 3.2 Detail: NWA 6259 Meteorite Mössbauer Spectra

**Figure 2:**
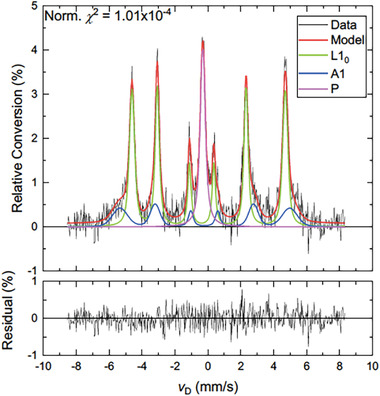
Mössbauer spectrum, fit and residual (X‐ray yield) obtained from the NWA 6259 meteorite. This spectrum is dominated by the L1_0_ phase (green trace) at 64.6 vol.%, with the balance occupied by the disordered A1 phase (blue trace) at 16.3 vol.% and an unidentified phase that is paramagnetic phase at room temperature at 19.1 vol.% (purple trace). The accompanying residual (bottom graph) representing the difference between the model and the data showing only small and non‐systematic discrepancies.

**Figure** **2** displays a representative Mössbauer spectrum, fit and residual (X‐ray yield) of phases present in the NWA 6259 meteorite specimen. The solid line (the red trace, referred to as the “Model”) drawn through the data points represents the least‐squares fit of the magnetic hyperfine pattern to the experimental data points, illustrated by the black trace. The maximum relative Mössbauer conversion percentage of ≈4.5% is in alignment with the large volume of specimen that was sampled and with the atomic concentration of iron in the meteorite (>50%). As a point of reference, a clean (i.e., unoxidized) surface of a thick *α*‐Fe sample demonstrates a peak conversion rate of ≈9.2%, at the same angle of incidence (in X‐ray yield). The fit of the model to the data is good, with a normalized *χ* ^2^ value of 1.01 × 10^−4^. The NWA 6259 Mössbauer spectrum is dominated by the signal from the atomically ordered L1_0_ phase (green trace) at 64.6 vol.%, with the balance occupied by the disordered A1 (fcc) phase (blue trace) at 16.3 vol.% and an unidentified phase of 19.1 vol.% (referred to as “P” in the graph legend, purple trace) that is paramagnetic at room temperature. The absolute abundance of these three iron‐containing phases varies with depth beneath the roughly polished surface of the meteoritic slab. The interior sample region (depth > 25–30 µm) is found to be enriched in the L1_0_ phase and depleted in the unidentified paramagnetic (P) phase, with phase percentage estimates close to 72% L1_0_‐type phase, 15% A1‐type phase and 13% of the paramagnetic phase, based on analyses of the γ‐ray data obtained at varied angles of incidence. **Table** **2** provides the refined Mössbauer parameters obtained from the NWA 6259 meteorite slab.

**Table 2:** Refined Mössbauer fitting parameters for the NWA 6259 meteorite slab, with the parameters listed using their usual symbols: abundance *A*, linewidth *Γ*, Gaussian distribution in hyperfine field *σ*, isomer shift *δ*, quadrupole splitting Δ, average hyperfine field *B*_hf_ and angle between the average remnant magnetization and the direction of the incoming photons *Θ*, respectively.

Phase∖Param.	*A* [%]	*Γ* [mm ^−1^s]	*σ* [mm ^−1^s]	*δ* [mm ^−1^s]	[Δ (mm ^−1^s]	*B*_hf_ [T]	*Θ* [rad]
L1_0_	64.55	0.193	0.205	0.178	0.426	28.89	1.061
A1	16.34	0.125	0.618	0.180	0.096	31.07	1.076
Paramagnetic	19.11	0.215	0.600	0.313	0.162	0	0.843

### 3.3 Detail: Processed FeNi Sample Mössbauer Spectra

**Figure 3:**
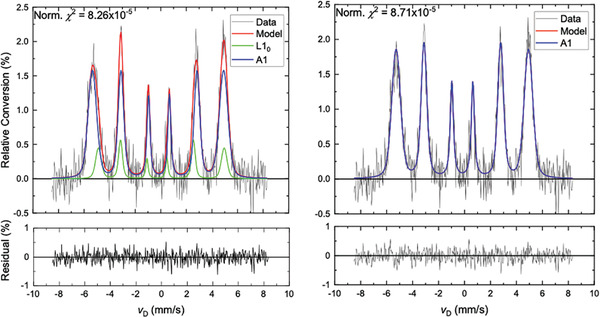
Mössbauer spectra obtained from a magnetic‐ and stress‐field annealed sample of Fe^62^Ni, fitted with a combination of A1 and L1_0_ phases (left panel) and with only the A1 phase (right panel). The improvement in the model fit upon inclusion of the ordered L1_0_ phase is quantified by the normalized χ^2^ value which is reduced from of 8.71 × 10^−5^ to 8.26 × 10^−5^ upon addition of the L1_0_ component spectrum.

Identification and quantification of phases present in the processed FeNi samples were achieved using the hyperfine Mössbauer parameters measured from the disordered A1 and the atomically ordered L1_0_ phases within the NWA 6259 meteorite (Figure 2, Table 2) as a calibration set. It is noted that the maximum relative conversion percentage of data obtained from all synthetic samples is reduced by a factor of two from that obtained from the meteorite, consistent with the smaller amount of material available to be probed and the larger relative linewidths observed that correspond to the lower degree of local atomic order. **Figure** **3** features a spectrum obtained from the magnetic‐field‐/stress‐field‐processed (FSA) Fe^62^Ni sample; this spectrum is highly representative of those obtained from all the synthetic specimens of this study and possesses the best signal‐to‐noise ratio among the acquisitions. It can be seen that an accurate fit to these Fe^62^Ni data requires introduction of the spectrum of the atomically ordered L1_0_ phase, unequivocally confirming the presence of tetrataenite in this spectrum. The appropriateness of including the ordered L1_0_ phase is quantified by the normalized χ^2^ value which improves from 8.71 × 10^−5^ to 8.26 × 10^−5^ upon addition of the L1_0_ spectral component. Further, the residual signal (bottom graphs) representing the difference between the model and the data has likewise improved, smoothing out the undulated residual background consistent with a better fit of the model to the data.

**Figure 4:**
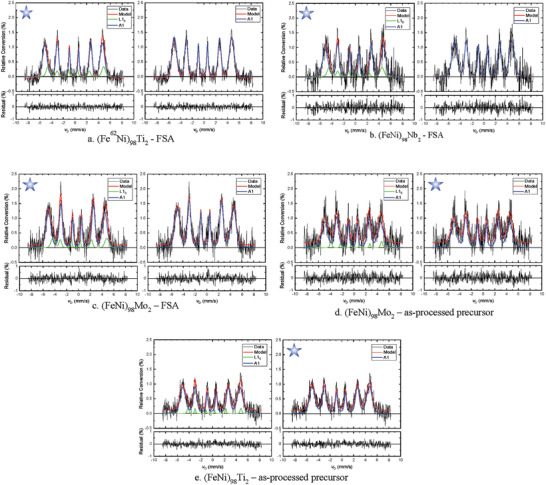
Mössbauer spectra obtained from all specimens of this study, along with the outcomes of fitting the two different models discussed in the text. The acronym FSA refers to samples thermally treated under simultaneous applied magnetic field and stress, while the as‐processed precursor refers to specimens in their original cold‐rolled state. Left‐hand‐side graphs display the fit to the model that contains both the L1_0_ (atomically ordered) and the A1 (atomically disordered) phases. Right‐hand‐side graphs display the fit to the model that contains only the A1 phase. The normalized χ^2^ values associated with each model fit, for each specimen, are listed in Table 3. The blue stars identify the most appropriate fit to the experimental data, in accordance with the values of the normalized χ^2^ parameters, the residual of the fit as well as with assessment of the presence of non‐physical spectra returned by the specific fitting model. In summary, the presence of the L1_0_ phase is statistically justified in all FSA samples and is not justified in all as‐processed precursor samples. Quantification of the phase fractions returned from these analyses is reported in Table 1.

Spectra and fitted models of all other samples investigated in this work are displayed in **Figure** **4**: there are three additional magnetic field‐stress annealed (FSA) FeNi‐based specimens and two as‐processed precursor specimens. In each of the five spectral pairs of Figure 3, the left‐side spectrum illustrates the fit to the model that includes both atomically ordered L1_0_ and disordered A1 components, whereas the right‐side spectrum contains the fit to the model containing only the disordered A1 phase. It is noted that the spectra of all of FSA‐processed samples cannot be fully interpreted within a model containing only the atomically disordered A1 phase; inclusion of the L1_0_ phase is necessary for convergence. In contrast, spectra obtained from the as‐processed precursor specimens may be fit without introduction of an additional L1_0_ phase; these data regress to the expected normally distributed Gaussian shot noise level *σ_G_* with *σ_G_* ∼ *n*_0_^−0.5^, where *n*_0_ is the average number of photons detected in each of the 512 individual velocity bins. For example, the data with the best available SNR, obtained from the Fe^62^Ni FSA‐processed sample, cannot be regressed below *σ_G_* = 1.6 × 10^−3^ (while *n*_0_^−0.5^ = 1.6 × 10^−3^) without the introduction of the L1_0_ phase.

While the amount of L1_0_‐type locally ordered phase determined in the FSA‐processed samples varies from specimen to specimen, with mean values in the range 16–22 vol.% (Table 1), none of the as‐processed precursor specimens contain significant evidence of ordered FeNi, tetrataenite. This conclusion is supported by the information in **Table** **3** that presents the goodness‐of‐fit *χ* ^2^ parameters resulting from application of these two models to the spectral data. In addition to statistically insignificant differences in the fits between the two models for data obtained from conventionally annealed specimens, the action of forcing a fit to the model that includes the L1_0_ phase returns Mössbauer signatures of unphysically large quadrupole splitting and isomer shift. These unphysical features may be seen in largely distorted L1_0_ components of the corresponding fits in Figure 3 for the conventionally annealed samples (green traces).

**Table 3:** Normalized goodness‐of‐fit *χ* ^2^ parameters resulting from application of the two models, described in the text, to the Mössbauer spectral data obtained from all synthetic samples of this study and displayed in Figure 4. “FSA” identifies the processing condition of annealing under simultaneous applied magnetic field and stress conditions; “precursor” refers to refers to specimens in their original cold‐rolled state.

Synthesized sample composition (atomic basis)	Annealing condition	Normalized χ^2^ parameters resulting from fit to model (errors in the last digit printed)		Comments
		Model contains L1_0_ & A1 phases	Model contains only A1 phase	
Fe^62^Ni	FSA	8.26 × 10^−5^	8.71 × 10^−5^	The model containing both L1_0_ and A1 phases applied to FSA sample data is statistically justified, relative to the model containing only the A1 phase.
(Fe^62^Ni)_98_Ti_2_	FSA	6.87 × 10^−5^	7.03 × 10^−5^	
(FeNi)_98_Nb_2_	FSA	1.3 × 10^−4^	1.3 × 10^−4^	
(FeNi)_98_Mo_2_	FSA	1.1 × 10^−4^	1.1 × 10^−4^	
(FeNi)_98_Mo_2_	Precursor	1.3 × 10^−4^	1.3 × 10^−4^	The model containing only the A1 phase applied to convention‐ally annealed data is the only statistically justified model; further, the model containing both Al and L1_0_ phases converge on unphysical Mössbauer signatures.
(FeNi)_98_Ti_2_	Precursor	7.6 × 10^−4^	7.7 × 10^−4^	

**Figure 5:**
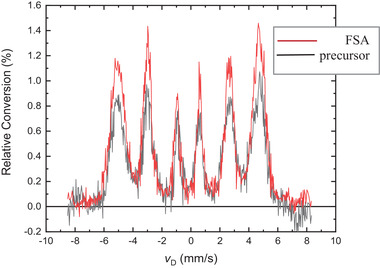
Direct comparison of spectra obtained from two separate samples of composition (Fe^62^Ni)_98_Ti_2_: one sample was studied in its magnetic field‐stress annealing (FSA) state and the other was studied in its cold‐rolled precursor state. The sharper, better‐formed Mössbauer peaks of the FSA‐treated sample are consistent with a higher degree of crystallinity as compared to that present in the precursor.

**Figure** **5** provides a direct comparison of spectra obtained from a FSA‐processed sample and the corresponding precursor of the same composition ((Fe^62^Ni)_98_Ti_2_); the amount of material probed in each case was the same. The difference in these two spectra is pronounced: the FSA spectrum evinces sharp and well‐formed Mössbauer absorption peaks relative to those of the precursor sample spectrum. The sharper peaks are consistent with a higher degree of crystallinity brought about by FSA thermal treatment. Thus, not only does magnetic‐field/stress thermal treatment promote the formation of the L1_0_ phase in FeNi alloys, but it also enhances crystallinity.

While the overall signal‐to‐noise ratios of these data are low, a feature that is a consequence of the wide distribution of hyperfine fields displayed by the synthetic samples and of the constrained intensity of the Mössbauer source utilized in this study, they are still statistically robust. A statistical uncertainty described by a Jacobian matrix provides an estimate of 20–90% relative to the value itself. This assessment places the lowest (i.e., the most pessimistic) position of the 95% confidence interval of the amount of L1_0_ phase present in the FSA‐processed FeNi samples at 2 vol.%. In other words, the very smallest confirmed amount of L1_0_‐ordered phase present in the FSA‐processed FeNi samples is 2 vol% while zero L1_0_‐ordered phase is present in the precursors. Correspondingly, the highest (i.e., the least pessimistic) determination of L1_0_‐ordered phase in the FSA‐processed samples is 27 vol%.

The average value of the resolved hyperfine fields *B*_hf_ for the L1_0_ phase in the artificial samples and in the natural sample are 29.7 +/− 0.4 and 28.9 +/− 0.4 T, respectively, while the values of *B*_hf_ for the A1 phase in these two samples are also very close, at 30.5 and 31.0 T, respectively (both within an error of +/− 0.4 T). The similarity of these values provides confidence in the phase identification for the artificial samples. However, the widths of these hyperfine fields (Δ*B*_hf_ = *σ*) are significantly different, with Δ*B*_hf_ (FSA samples) > Δ*B*_hf_ (meteorite). Examples of comparisons of the widths of the determined hyperfine fields are *σ* (L1_0_, NWA) = 0.21 T, *σ* (L1_0_, FSA) = 0.35 T, and *σ* (A1, NWA) = 0.62 T, *σ* (A1, FSA) = 0.68 T. Thus it may be concluded that the degree of L1_0_‐type chemical order present in the processed samples is substantially lower than that of the natural meteorite sample, Figures 1, 2 and **Table** **4**.

**Table 4:** Refined Mössbauer fitting parameters for the Fe^62^Ni (FSA) artificial sample, with the parameters listed using their usual symbols.

Phase∖Param.	*A* [%]	*Γ* [mm ^−1^s]	*σ* [mm ^−1^s]	*δ* [mm ^−1^s]	*Δ* [mm ^−1^s]	*B*_hf_ [T]	*Θ* [rad]
L1_0_	22.26	0.150	0.351	0.151	0.354	30.83	1.043
A1	77.74	0.152	0.547	0.184	−0.090	31.87	0.976

The average values of the quadrupole splitting for the L1_0_ phase, representing the distortion of the local atomic environment, is 0.48 mm^−1^ s for the meteorite and 0.36 mm^−1^ s for the FSA‐processed sample, while the corresponding values for the A1 phases in all samples are close to zero, consistent with the cubic local atomic symmetry. These outcomes imply that the L1_0_ phase suffers from more local disorder in the artificial samples than in the natural meteoritic sample. Additionally, Figure 4 shows that the Mössbauer absorption peaks associated with the precursor specimen are significantly broader than those of the FSA specimen and are of lower intensity, despite the fact that the same amount of sample was probed in both experiments. The sharper, better‐formed Mössbauer peaks of the FSA‐treated sample are consistent with a higher degree of crystallinity as compared to that present in the precursor sample, indicating that the FSA process not only promotes formation of the L1_0_ phase but also amplifies overall crystallinity.

## 4 Conclusion

This work confirms the presence of atomic order in nominally equiatomic iron‐nickel‐based bulk alloys that has been induced via application of magnetic field and stress during 6 weeks of mild thermal treatment. These results not only challenge conventional understanding of the kinetic processes underlying the formation of the extraterrestrial mineral tetrataenite but also inform the perspective of new additions to the advanced permanent magnet portfolio for next‐generation green technologies.

Tetrataenite's magnetic properties have been considered as a record of the paleomagnetic fields present in the early solar system.^[^ ^45^ ^]^ Indeed, conclusions and questions arising from the study of metallic meteorites motivate, in part, NASA's 2023 Psyche mission to explore a unique nickel–iron metal asteroid that is hypothesized to contain clues of the building blocks of our solar system.^[^ ^46^ ^]^ The planetary science community has made extensive use of Mössbauer spectroscopy for the detection of L1_0_ FeNi in selected meteorites^[^ ^43^, ^47^ ^]^ with essentially all studies performed at room temperature on thin cut slivers or on powdered samples. In this current work, spectra obtained from a macroscopic slab of the NWA 6259 meteorite using different incident photon angles indicate that the relative amount of tetrataenite increases at the expense of the paramagnetic iron phase by as much as 10% as the sampling depth increases, from a surface value of 60% to an interior value of 70%. This observation strongly suggests that surface artifacts, perhaps introduced during mechanical polishing or due to oxidation, impact the nature of the phases contained in this meteorite. Accordingly, conclusions regarding phase identity and atomic ordering conditions derived from Mössbauer spectroscopy data obtained from other metallic meteorites may warrant re‐examination and re‐evaluation.^[^ ^48^, ^49^ ^]^

Results from this study also contribute new knowledge regarding the development of new types of advanced permanent magnets to address supply chain issues and to more broadly support the development of green technologies. The unequivocal confirmation of induced tetragonality as well as of chemical order in bulk iron‐nickel materials highlights pathways to form tetrataenite in industrially relevant timeframes as well as to increase its magnetocrystalline anisotropy energy. The low value of magnetocrystalline anisotropy measured from the processed systems of this study is consistent with a low degree of induced atomic order. Notably, the materials of this current study do not contain large amounts of metalloid alloying additions, such as phosphorus and carbon, as were utilized in other works that reported synthesis of tetrataenite.^[^ ^18^, ^50^, ^51^ ^]^ Preliminarily speaking, the combination of stress and magnetic field applied in‐situ during thermal treatment has accomplished in 6 weeks what Nature requires millions of years to achieve. As the lattice diffusivity of the FeNi equiatomic composition has been reported as ≈1 × 10^−27^ cm^2^ s^−1^ at *T* = 573 K,^[^ ^52^ ^]^ the process utilized in this work is estimated to accelerate diffusion by ten or more orders of magnitude. Additionally, it is known that crystallographic texture, or alignment, is also produced in FeNi alloys subjected to SFA processing.^[^ ^31^, ^34^ ^]^ In addition to magnetocrystalline anisotropy, crystallographic alignment is an important engineering parameter necessary to optimize the strength (i.e., maximum energy product) of a permanent magnet.

While indeed a unique and indispensable tool, Mössbauer spectroscopy does not provide information on the extent, dimensions or distributions of identified magnetic phases. Given the conclusion that L1_0_‐type atomic order is much better developed in the NWA 6259 meteorite than in the processed samples, as furnished by comparison of the measured hyperfine magnetic field *B*_hf_ distributions, it is not surprising that the magnetocrystalline anisotropy measured from the synthetic tetrataenite is low. At the current time the precise effects of this combination magnetic and strain energies to the Fe‐Ni thermodynamic landscape are unclear, but preliminarily it is considered that these external energies alter the stability of the exchange‐split 3d bands and impact the electronic free energy of the system. The magnitude of the magnetic field applied during the thermal treatment step is much too small to produce an appreciable Zeeman energy; therefore, some other factors must be in play.

The outcomes of this work invite contemplation of further refinements to processing protocols to maximize the fraction of well‐ordered, bulk synthetic tetrataenite. It is considered that the confirmed L1_0_‐type regions within the A1 matrix may now be able to serve as “seeds” for continued growth of the ordered phase, in a crystallographically oriented fashion. Additionally, an increased time of annealing, beyond the 6 weeks employed in this study, is also expected to amplify the fraction of atomically ordered phase. For example, the introduction of large amplitude ultrasonic excitation may be able to promote ultrasonic‐assisted diffusion.^[^ ^53^ ^]^ Modifications to the mode of in‐situ magnetic field application during annealing are ongoing.

## 5 Experimental Section

### Samples and Processing

Both synthetic and natural samples were examined in this work. A comprehensive description of the samples’ synthesis, processing, and characterization history as well as details concerning the mode of magnetic‐ and stress‐field annealing are provided in refs. [31, 34] Briefly, bulk equiatomic FeNi‐based polycrystalline alloy samples were obtained from molten elemental sources and homogenized at 500 °C for 100 h. Small amounts (2 at%) of Ti, Mo, and Nb were originally added to some compositions with the intent to influence the thermodynamic stability of the FeNi phases within the alloy; however, there was currently no support for this hypothesis. Additionally, some compositions were synthesized with the isotope ^62^Ni for neutron diffraction students. None of these compositional variations were considered to have influenced the results reported here. After solidification and homogenization, samples were subsequently subjected to severe mechanical deformation by cold‐rolling to form elongated, flattened strips of approximate dimensions 5 cm × 1 cm × 0.3 cm with an approximate strain level *ε* in the range 85–95%. A subset of these deformed strips was mounted on the customized furnace sample assembly for thermal treatment in an inert atmosphere under the simultaneous action of applied tensile stress (≈6.2 MPA) and static uniform magnetic field (≈0.7 T at elevated temperature). These magnetic‐ and stress‐field processed samples were identified by the acronym “FSA” (field‐stress annealed). Specimens were heated above the reported 320 °C order‐disorder temperature of L1_0_ FeNi, held for 5 min, and then slowly cooled to T≈285 °C at a rate of 0.1 K min^−1^ and held for 48 days prior to furnace cooling to room temperature. For comparison purposes, naturally formed tetrataenite as present in the NWA 6259 meteorite was also studied. This Ni‐rich a taxite meteorite holds the distinction of being the second‐highest Ni content meteorite yet discovered (>42% net.^[^ ^54^ ^]^) and possesses the largest relative amount of NiFe L1_0_ phase observed to date in meteorites. It contained small FeS inclusions and was dominated by a well‐developed tetrataenite main phase of composition Fe_55_Ni_45_.^[^ ^55^, ^56^ ^]^ The meteorite sample was in the form of a roughly polished slab of approximate dimensions 65 mm × 35 mm and thickness of 4 mm.

### Mössbauer Spectroscopic Investigation

The ^57^Fe Mössbauer spectra were collected from pieces of alloy strips that were assembled into a flat mosaic arrangement to provide maximal area for collection (1–2 cm^2^). Spectra were obtained at angles of incidence ranging from 5 to 90°, with optimal X‐ray sensitivity obtained at angles in the range 30–45°. These strips were examined in their as‐received state, with no special surface preparation. Data from the NWA 6259 meteorite were collected from an area of ≈10 mm × ≈40 mm within the center section of the slab, in the same region probed in other studies of this specimen.^[^ ^54^, ^55^ ^]^ Experiments were conducted at room temperature in the backscattering geometry utilizing a ^57^Co (Rh) source of approximate activity of 5 mCi. The Doppler velocity modulation was performed in the constant acceleration mode, while the pulse acquisition was conducted using a Xe:CO_2_‐filled proportional detector, with a Be front window. Data acquisition was performed using a purpose‐built multi‐parameter analyzer, capable of recording energies of both the X‐ray and the γ‐quanta as well as documenting the instantaneous Doppler velocity. This configuration allowed for optimal post‐acquisition energy discrimination of two Mössbauer spectra–one spectrum for the conversion X‐rays (Fe─K, at ≈6.4 keV) and another for the backscattered γ‐rays (at ≈14.4 keV). An additional background spectrum was obtained by the post‐discrimination of the non‐resonant photons (≈16–200 keV) and used to correct for geometric (stearic angle of pickup) effects and Doppler drive distortions. The two collected spectra correspond to two different characteristic absorption lengths and, especially at oblique angles of incidence, allowed for depth discrimination of both structural and magnetic information. While the depth sensitivities of these two spectra were complicated functions of elemental composition, angle of incidence, sample thickness, etc., the general rule is that the X‐ray spectra were substantially more surface‐sensitive than were the γ‐ray spectra: the former probe a characteristic length in the range 1–10 µm while the latter originate from deeper within the sample, up to ≈50 microns. This type of data acquisition collects both backscattered γ‐rays and conversion X‐rays and is well‐suited to the investigation of a variety of samples, including thick samples which cannot be characterized in the conventional transmission geometry, relatively thin samples of large lateral area (e.g., thick films on a wafer) and samples with features that render the surface condition unrepresentative of the bulk interior region. More details on the digital electronics and software enabling this type of data acquisition are provided in the work of Borisov et al.^[^ ^57^ ^]^ and O'Brien et al.^[^ ^58^ ^]^

Optimal Mössbauer results were achieved with the use of low‐fluorescent‐background Perspex sample holders in combination with sufficiently thick (≈5 cm) primary beam Howitzer collimators. While a Mn absorption filter was incorporated to suppress the non‐resonant fluorescent background arising from samples’ high Ni content, it was found to provide no substantial improvement of the signal‐to‐noise ratio. Data were collected over a time period of 3–4 weeks per run with incidence angles ranging from 5 to 90° and were fit to a non‐linear least‐squares routine using custom‐created software, allowing for full‐Hamiltonian hyperfine parameter modelling and the inclusion of hyperfine field and quadrupole moment distributions. Mössbauer parameters were first determined for phases present in the natural meteorite NWA 6259 specimen; these hyperfine parameters were then used as a reference to analyze spectra obtained from the synthetic FeNi samples.

### Magnetic Characterization

Magnetic characterization was performed on the processed sample strip pieces by means of vector vibrating sample magnetometry (VVSM), using a custom‐build instrument^[^ ^59^ ^]^ and single‐axis SQUID magnetometry (MPMS XL5, Quantum Design, CA). Rotational VVSM scans were performed at room temperature in applied fields up to *µ*_0_ *H* = 2 T, with both *m_x_* and *m_y_* in‐plane components of the magnetization recorded for the deduction of the lattice torque.

### Statistical Analysis—Pre‐Processing of Data

The data pre‐processing, for all Mössbauer spectra presented here, followed the following standardized algorithm: the unfiltered raw detector data stream (timestamp, Doppler velocity, photon energy) was cast into 2D data matrices (number of detected photons as a function of Doppler velocity and photon energy) without any filtering. The resulting matrices were averaged into two specific energy bands, corresponding to back‐scattered gamma photons and conversion X‐rays, using optimal energy discrimination, as detailed in Ref. [58] The spectral normalization was done using the non‐resonant background of photons detected with energies above both resonant absorption lines, and very efficiently corrects for the variation of stearic angle of pick‐up as a function of sample‐to‐source distance. No further processing was needed ‐, i.e. no background subtraction or removal of outliers were performed.

### Statistical Analysis—Data Presentation

The datasets for the individual samples studied had been acquired in a random sequence and had been processed completely independently from each other. The fits to these two data products were performed using a general χ^2^ minimization procedure, with initial guess values cast on a regular grid for all fitting parameters. The convergence criterion used was the location of the global minimum of χ^2^ to the limit of the normally distributed noise in all dimensions of the fitting parameters’ space.

### Statistical Analysis—Statistical Methods Used

The errors of the fitting parameters were computed using the standard approach as the square root of the diagonal elements of the product of the covariance matrix and the mean‐square error (itself a function of the minimal χ^2^ value, at convergence) and was further corrected with the Student's *t*‐distribution function, for the exact number of elements in the data vector, using a confidence band of 95%. The evaluation of the covariance matrix relies on the iterative computation of the Jacobian matrix, every element of which was converged to a relative uncertainty of 0.3%.

### Statistical Analysis—Software

The error evaluation described above was performed using a custom‐developed software, which was available from the Authors upon reasonable request.

## Conflict of Interest

The authors declare no conflict of interest.

## Data Availability

The data that support the findings of this study are available from the corresponding author upon reasonable request.
